# Community Rates of Alzheimer’s Disease and Related Dementias and Supportive Services in CT, MA, & RI

**DOI:** 10.1093/geroni/igaf122.2519

**Published:** 2025-12-31

**Authors:** Michelle Ward, Dongfang Hong, Nachalie Rodriguez-Cruz, Mengshi Liu, Elizabeth Dugan

**Affiliations:** University of Massachusetts Boston, Boston, Massachusetts, United States; University of Massachusetts Boston, Boston, Massachusetts, United States; University of Massachusetts Boston, Boston, Massachusetts, United States; University of Massachusetts Boston, Boston, Massachusetts, United States; University of Massachusetts Boston, Boston, Massachusetts, United States

## Abstract

The consequences of Alzheimer’s Disease and Related Dementias (ADRD) are well documented, but beyond the individual, family, and health system costs are implications for communities that are not well-studied. The aim of this study is to describe community rates of ADRD and the per capita rate of supportive services (caregiver support groups and adult day health services). Data were obtained from the 2025 Healthy Aging Data Reports (healthyagingdatareports.org) for CT, MA, and RI. Small area estimation techniques were used to calculate age- and sex-adjusted rates of ADRD for Medicare fee-for-service beneficiaries ever diagnosed (2020-2021). Caregiver support groups were obtained from the Alzheimer’s Association (2023). Adult day service data sources were the CT Association of Adult Day Services, MA EOHHS, and RI Dept. of Health. Average state rates in adults 65+ were: CT 13.9% (Sherman, 6.9%; Hartford, 22.4%); MA 12.9% (Washington, 6.7%; Chelsea, 20.0%); and RI 12.0% (Jamestown, 7.3%; Central Falls, 16.3%). Per capita, RI (n = 15.9) had the most adult day centers per 100,000 adults age 65+, followed by MA (n = 12.0) and CT (n = 6.0). For ADRD caregiver support groups, CT and RI (n = 4.6) had the most groups per 100,000 older adults, followed by MA (n = 2.1). The rates of ADRD vary slightly by state, with larger variations within each state. Many communities with higher rates of ADRD do not have any caregiver support groups or adult day programs, which provide crucial respite and emotional care. Understanding disparities in access to ADRD services will enable communities to identify needs in increasing supportive services.

